# Supplementary material for: Transcriptome Analysis Identifies the Crosstalk between Dendritic and Natural Killer Cells in Human Cutaneous Leishmaniasis
Source: Microorganisms. 2023 Jul 29;11(8):1937. doi: 10.3390/microorganisms11081937 (PMC10459107; doi:10.3390/microorganisms11081937)
Supplement: Supplementary file 1 [file microorganisms-11-01937-s001.zip › Figure S1.pdf]

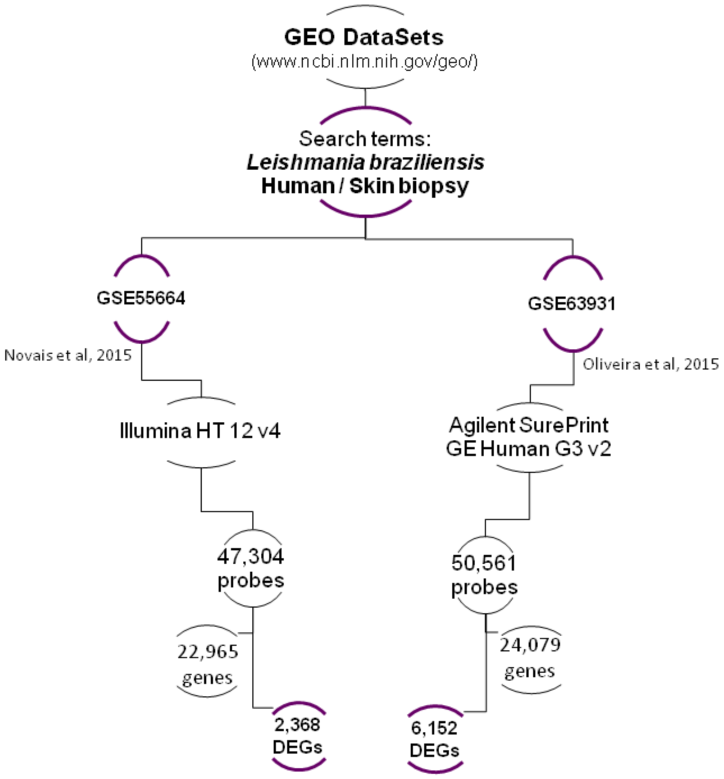

Supplementary Figure S1. Identification of Differentially Expressed Genes in Human Cutaneous Leishmaniasis using publicly available gene expression datasets. Search strategy to identify datasets containing raw gene expression data from the analysis of human skin biopsies of localized cutaneous leishmaniasis caused by Leishmania braziliensis (obtained from the public data repository Gene Expression Omnibus - www.ncbi.nlm.nih.gov/geo/). Two transcriptomes were identified: GSE55664 (Illumina HT 12 v4 platform) and GSE63931 (Agilent SurePrint GE Human G3v2 platform), containing 47,304 probes and 50,561 probes, respectively. Considering the absolute number of genes in each dataset (22,965 and 24,079), a total of 20,940 were present in both transcriptomes, leading to 2,368 and 6,152 Differentially Expressed Genes (DEGs), respectively.
